# Development of Solid Nanosystem for Delivery of Chlorhexidine with Increased Antimicrobial Activity and Decreased Cytotoxicity: Characterization and In Vitro and In Ovo Toxicological Screening

**DOI:** 10.3390/molecules30010162

**Published:** 2025-01-03

**Authors:** Alexandra-Ioana Dănilă, Mihai Romînu, Krisztina Munteanu, Elena-Alina Moacă, Andreea Geamantan-Sîrbu, Iustin Olariu, Diana Marian, Teodora Olariu, Ioana-Cristina Talpoş-Niculescu, Raluca Mioara Cosoroabă, Ramona Popovici, Ştefania Dinu

**Affiliations:** 1 Faculty of Medicine, Victor Babes University of Medicine and Pharmacy, 300041 Timisoara, Romania; alexandra.danila@umft.ro (A.-I.D.); krisztina.munteanu@umft.ro (K.M.); 2 Research Center of Digital and Advanced Technique for Endodontic, Restorative and Prosthetic Treatment (TADERP), Victor Babes University of Medicine and Pharmacy, 300041 Timisoara, Romania; rominu.mihai@umft.ro; 3 Faculty of Dental Medicine, Victor Babes University of Medicine and Pharmacy, 300041 Timisoara, Romania; ioana.talpos-niculescu@umft.ro (I.-C.T.-N.); raluca.cosoroaba@gmail.com (R.M.C.); ramona.popovici@umft.ro (R.P.); dinu.stefania@umft.ro (Ş.D.); 4 Faculty of Pharmacy, Victor Babes University of Medicine and Pharmacy, 300041 Timisoara, Romania; alina.moaca@umft.ro (E.-A.M.);; 5 Research Centre for Pharmaco-Toxicological Evaluation (FARMTOX), Victor Babes University of Medicine and Pharmacy, 300041 Timisoara, Romania; 6 Faculty of Dental Medicine, Vasile Goldiş Western University of Arad, 310414 Arad, Romania; marian.diana@uvvg.ro; 7 Faculty of Medicine, Vasile Goldiş Western University of Arad, 310414 Arad, Romania; olariu.teodora@uvvg.ro; 8 Pediatric Dentistry Research Center, Victor Babes University of Medicine and Pharmacy, 300041 Timisoara, Romania

**Keywords:** maghemite, chlorhexidine digluconate solution, human immortalized keratinocytes, murine epidermal cells, in vitro and in ovo screening, irritant potential, apoptotic index

## Abstract

The evaluation of chlorhexidine-carrier nanosystems based on iron oxide magnetic nanoparticles (IOMNPs), has gained significant attention in recent years due to the unique properties of the magnetic nanoparticles (NPSs). Chlorhexidine (CHX), a well-established antimicrobial agent, has been widely used in medical applications, including oral hygiene and surgical antisepsis. This study aims to report an in vitro and in ovo toxicological screening of the synthesized CHX-NPS nanosystem, of the carrier matrix (maghemite NPSs) and of the drug to be delivered (CHX solution), by employing two types of cell lines—HaCaT immortalized human keratinocytes and JB6 Cl 41-5a murine epidermal cells. After the characterization of the CHX-NPS nanosystem through infrared spectroscopy and electronic microscopy, the in vitro results showed that the CHX antimicrobial efficacy was enhanced when delivered through a nanoscale system, with improved bioavailability and reduced toxicity when this was tested as the newly CHX-NPS nanosystem. The in ovo screening exhibited that the CHX-NPS nanosystem did not cause any sign of irritation on the chorioallantoic membrane vasculature and was classified as a non-irritant substance. Despite this, future research should focus on optimizing this type of nanosystem and conducting comprehensive in vivo studies to validate its therapeutic efficacy and safety in clinical settings.

## 1. Introduction

Chlorhexidine (CHX) is the most commonly used antiseptic worldwide, being considered the reference antiseptic treatment in dentistry and oral healthcare infections, due to its potent antimicrobial properties [1,2]. Its chemical structure consists of two 4-chlorophenyl rings linked by a central hexamethylene chain, contributing to its high alkalinity and positive charge, and facilitating its interaction with negatively charged bacterial cell walls. This interaction leads to disrupting bacterial cell membranes, resulting in cell lysis and death, making CHX effective against a broad spectrum of microorganisms, including Gram-positive and Gram-negative bacteria, fungi, and some viruses [3]. The main clinical application of CHX is in dental practice to reduce dental plaque formation, for gingivitis and periodontitis prevention and control, for root canal treatment, in different stages of dental implants, and in oral surgery and oral medicine (as an antiseptic agent) [4], yet several lines of evidence still suggest its effectiveness in surgery for skin disinfection and preoperative whole-body disinfection and as a treatment for wounds or burns [5,6]. The use of CHX in conjunction with tooth brushing reduces plaque formation and minimizes the risk of pathogen colonization in the oral cavity, leading to superior oral hygiene compared to that from tooth brushing alone [7,8]. It has been stated that CHX mouth rinses, particularly at concentrations of 0.12% to 0.2%, significantly reduce oropharyngeal bacterial colonization [9,10]. In addition, CHX oral care could reduce the incidence of ventilator-associated pneumonia (VAP) by approximately 40% in mechanically ventilated patients, highlighting its importance in intensive care unit (ICU) protocols [10,11]. CHX is beneficial in managing oral mucositis, particularly in patients undergoing chemotherapy [12,13]. This is particularly relevant as oral mucositis can lead to severe complications, including increased risk of infections and prolonged hospital stays; therefore, CHX’s efficacy is attributed to its ability to inhibit bacterial growth and promote oral hygiene, thereby reducing the inflammatory response associated with mucositis [12,13].

Although CHX was considered a gold standard antimicrobial and has been used as a therapeutic agent since the 1950s, the use of CHX is not without controversy, particularly regarding its long-term application in ICU settings. Acıkan et al. [14] highlighted the cytotoxic effects of CHX on macrophages, demonstrating that even low concentrations can lead to necrosis and DNA damage, reinforcing CHX’s risk of genotoxicity across various organ systems. Moreover, Madrazo-Jiménez et al. [15] demonstrated that CHX, when applied topically, can affect the wound healing process in Wistar rats, indicating potential systemic effects beyond local application. Some studies have also raised concerns about potential adverse effects, including the development of resistance and alterations in the oral microbiome [16,17]. Additionally, there is an ongoing debate about the optimal protocols for CHX use, including the concentration and frequency of application. For example, while some guidelines recommend daily CHX rinses, others suggest that a more tailored approach may be necessary to balance efficacy with safety [16,17]. However, the effectiveness of CHX can be influenced by various factors, including the concentration used and the duration of application [9,18]. Other drawbacks that limit CHX use include the stability of this molecule (its primary degradation product is p-chloroaniline, a compound known to be hemotoxic and carcinogenic) [19] and its inactivation in body fluid [5]. Despite its widespread use, the development of standardized oral care bundles that incorporate CHX may enhance compliance and improve patient outcomes [18,20]. Given the aspects presented above, finding novel alternatives aiming to augment CHX’s therapeutic potential and minimize side effects has become an imperative matter.

The use of nanotechnology to manufacture delivery nanoplatforms for CHX still represents an attractive option. So far, several options have been proposed as drug delivery nanosystems for CHX with promising results, such as iron oxide magnetic nanoparticles (IONMPs) coated with chitosan [21,22], chlorhexidine liposomes [23], chitosan-coated liposomes [24], amino silane-coated magnetic nanoparticles functionalized with chlorhexidine [5], or silver nanoparticles with chlorhexidine [25], but further studies are required for the clinical use of these nanoscale delivery systems.

In recent years, iron oxide magnetic nanoparticles (IOMNPs), in particular magnetite (Fe_3_O_4_) and maghemite (γ-Fe_2_O_3_), represent the most studied FDA-approved nanomaterials for biomedical applications due to their distinctive features as biocompatibility, physicochemical stability, and superparamagnetic behavior [26,27]. In addition, these materials have a large surface, which means that they can be relatively easier to conjugate or encapsulate, and dimensions of the order of nanometers, for which they allow an increased circulation and a precise distribution [28,29,30]. To be suitable for clinical use, magnetic nanoparticles based on iron oxides must have, in addition to superparamagnetic behavior, a pure chemical composition and small dimensions [31]. In the biomedical field, IOMNPs have been extensively studied for their potential in drug delivery systems and cancer therapies. Their superparamagnetic nature allows for the targeted delivery of therapeutic agents to specific sites within the body, minimizing side effects on healthy tissues [32,33,34]. Therefore, iron oxide nanoparticles (IONPs) have emerged as a significant tool in both dentistry and medicine due to their unique properties and versatility. Their surface can be modified with various biocompatible polymers, surfactants, or other interest compounds, to enhance their stability and circulation time in the bloodstream [35,36,37,38,39]. This modification allows for the targeted delivery of therapeutic agents directly to diseased tissues, minimizing side effects and improving treatment efficacy [32,40]. Furthermore, the biocompatibility and low toxicity of IONPs make them suitable candidates for such applications, as they can be safely administered to patients [41,42]. In dentistry, IONPs have gained attraction for their applications in imaging and therapeutic interventions. Their superparamagnetic properties can be utilized in MRI to enhance the visualization of dental structures and pathologies, such as tumors or infections within the oral cavity [43,44]. Additionally, IONPs can be integrated into dental materials to improve their antibacterial activity, which is crucial for preventing infections and promoting healing in dental procedures [41,45]. Furthermore, the potential of IONPs in regenerative dentistry is being explored. Their ability to promote cell proliferation and differentiation makes them suitable for applications in tissue engineering and regenerative therapies. For instance, IONPs can be used to enhance the delivery of growth factors or stem cells to damaged dental tissues, facilitating repair and regeneration [46]. The magnetic properties of these nanoparticles also allow for the targeted delivery of therapeutic agents to specific sites within the oral cavity, improving treatment outcomes.

The antibacterial properties of IOMNPs have also been an interesting subject of research. Various studies have demonstrated their effectiveness against a range of bacterial strains, including *Escherichia coli* and *Staphylococcus aureus*, suggesting their potential use in treating bacterial infections and preventing biofilm formation on medical devices [47,48,49]. The mechanism behind this antibacterial activity is believed to involve the generation of reactive oxygen species (ROS) and the disruption of bacterial cell membranes, leading to cell death [47,50]. Furthermore, the ability of IOMNPs to inhibit biofilm formation makes them particularly valuable in clinical settings, where biofilm-associated infections pose significant challenges [48,50]. The FDA has approved several formulations of IOMNPs for clinical use, further supporting their safety profile [32,51]. Despite the promising applications of IOMNPs, concerns regarding their potential toxicity remain. Studies have indicated that exposure to IOMNPs can induce oxidative stress and apoptosis in certain cell types, raising questions about their long-term safety in biomedical applications [52,53]. It is essential to conduct thorough toxicological assessments to understand the implications of IOMNP exposure and to develop strategies to mitigate any adverse effects [33,53]. Ongoing research is essential to fully understand the long-term effects and potential risks associated with their use in both medical and dental applications.

Therefore, the current study aimed to obtain and investigate CHX encapsulated within the maghemite nanoparticles’ surface (CHX-NPS), to minimize CHX’s toxicity while maximizing its antimicrobial efficacy, by performing an in vitro and an in ovo toxicological screening, thus demonstrating the versatility of the obtained maghemite-nanoparticle-based nanosystem improving chlorhexidine bioavailability. It is well known that the nanoparticle’s surface modification or functionalization leads to increased delivery specificity [54,55]. In addition, the encapsulation of therapeutic agents within maghemite nanoparticles can be tailored to achieve a specific release profile. By adjusting the physicochemical features of the maghemite matrix, one can control the rate of the release, thus leading to the maintenance of the therapeutic levels of the attached agent over extended periods [56]. The in vitro models used in this study were two healthy cell lines: HaCaT immortalized human keratinocytes and JB6 Cl 41-5a murine epidermal cells at 24 h after their exposure to maghemite NPSs, CHX-NPS, and 2% CHX solution (a rather high concentration compared to those reported in the scientific literature), for short-term intervals. The in ovo screening was assessed using the HET-CAM test (Hen’s Egg Chorioallantoic Membrane) to investigate if the test samples showed any potential irritant effect on the chorioallantoic membranes of the hen’s egg. Therefore, based on the outcomes obtained, one can affirm if the nanosystem based on maghemite NPSs loaded with CHX exhibits safety use and biocompatibility with both healthy cell lines since CHX is widely used as an antiseptic in dental and cutaneous applications [57,58]. The CHX concentration used (2%) was chosen to be clinically relevant as a bacteriostatic and bactericidal agent [57].

## 2. Results

Following the obtained results, we will be able to state whether our synthesized nanosystem consisting of maghemite nanoparticles loaded with CHX on the surface presents a good biosafety profile for future in vivo studies. In this context, we performed a preliminary in vitro and in ovo toxicological screening to investigate if the synthesized nanosystem exhibited signs of toxicity on the healthy cell lines taken into account or signs of irritation on the chorioallantoic membrane of the hen’s egg.

### 2.1. Characterization of the Nanosystem Based on Maghemite-NPS-Loaded CHX (CHX-NPS)

#### 2.1.1. Fourier Transform Infrared Spectroscopy (FTIR) Investigation

**Figure 1:**
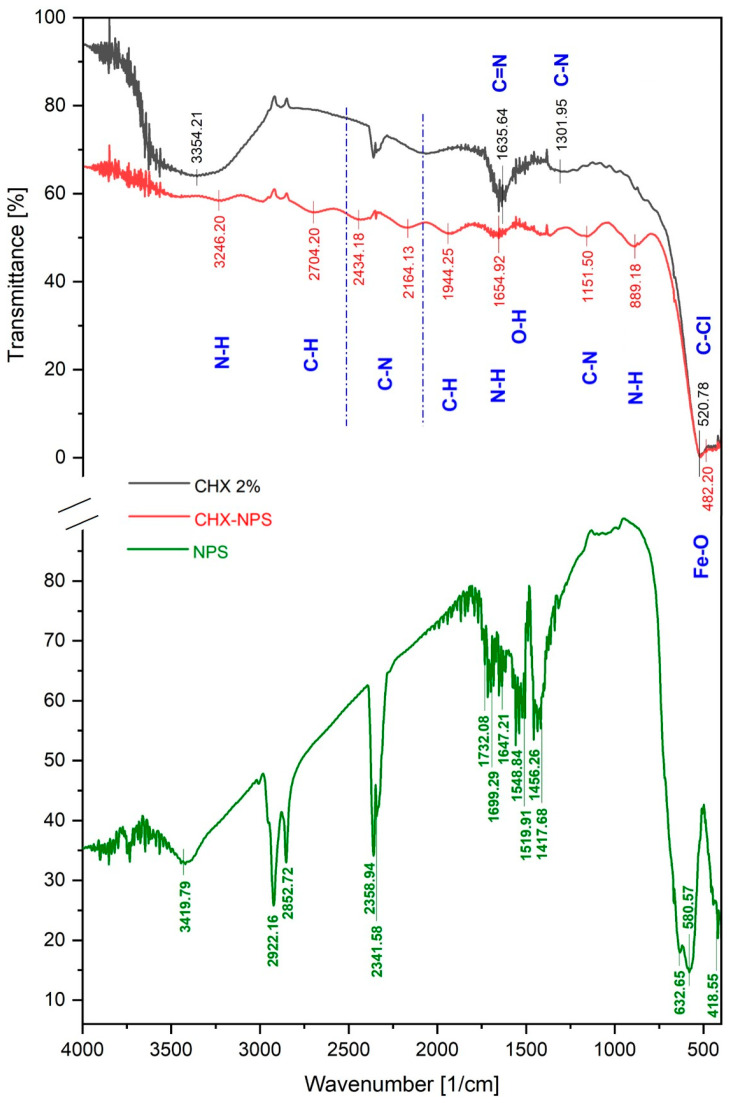
FTIR spectra of CHX solution (black line), naked maghemite NPSs (green line), and nanosystem CHX-NPS (red line).

Figure 1 shows the FTIR spectra of the 2% CHX solution, the naked maghemite NPSs, and the synthesized nanosystem CHX-NPS, obtained after the encapsulation of the CHX solution into the maghemite NPS surface. It is observed that surface modification of the maghemite nanoparticles with the CHX solution shifts the adsorption peaks to shorter wavelengths. The weak band recorded at 3246.20/cm in the case of the CHX-NPS nanosystem corresponds to the prominent N–H stretching vibrations from secondary amine groups present in the CHX solution.

A smaller absorption peak recorded at 3246.20/cm, in the case of the CHX-NPS nanosystem, is considered to be the result of the interaction between an overtone of the band recorded at 1654.92/cm with the symmetric N-H stretching band. The strong in-plane NH_2_ scissoring absorption at 1635.64/cm for the CHX solution and 1654.92/cm for the CHX-NPS nanosystem, as well as the out-of-plane wagging at 889.18/cm from the CHX-NPS nanosystem, are characteristic for the primary amines (N-H bending vibrations). The strong absorption band at 1635.64/cm from the CHX spectrum may be ascribed to the C=N stretching vibration, a characteristic of biguanide groups. At the same time, on the CHX-NPS spectrum, the weak signals recorded around 1600/cm can be attributed to the bending vibrations of hydroxyl functional groups from the surface of maghemite NPSs.

The C-N stretching absorption functional groups (assigned to the secondary amines) were recorded at 1301.95/cm (aromatic) for the CHX solution and at 1151.50/cm (aliphatic) for the CHX-NPS nanosystem. The absorbance spectrum for the CHX-NPS nanosystem presents a band recorded at 2164.13/cm that could be ascribed to the C=N stretching vibration functional groups, whereas the band recorded at 2434.18/cm is associated with the C-N stretching vibration functional group, which confirm the presence of CHX solution successfully attached to the maghemite NPS surface. Another confirmation of the attachment of CHX solution within the maghemite NPSs is represented by the absorption peak located at 520.78/cm, visible on both spectra, which highlights the presence of C-Cl stretching functional groups from the CHX solution, which is formed of chlorophenyl rings connected with the biguanide groups by a hexamethylene bridge.

The specific bands for iron oxide magnetic nanoparticles (IOMNPs) are located at 2704.20/cm recorded on the CHX-NPS spectrum, which can be assigned to the C-H stretching vibration from the triethylenetetramine compound, used as a reduction agent for the maghemite NPS synthesis, as well as at 1944.25/cm, which corresponds to the C-H functional group’s bending vibration, also characteristic of the triethylenetetramine compound. The triethylenetetramine compound is also visible on the maghemite NPS spectrum at 3419.79/cm, 2922.16/cm, and 2852.72/cm, assigned to the N-H stretching vibration from the amine functional groups, as well as to the peaks recorded between 1650 and 1580/cm, attributed to the N-H bending vibration from amine functional groups. Moreover, on the maghemite NPS spectrum, the C=N functional groups’ stretching vibration is recorded between 1690 and 1640/cm, and around 1400/cm the adsorption peaks are assigned to the C-H functional groups’ bending vibration. The last adsorption peaks on the maghemite NPS spectrum are located in the inorganic domain and correspond to the Fe-O stretching vibration from the iron oxide nanoparticles. The peak recorded at 480.20/cm on the CHX-NPS spectrum, associated with the Fe-O vibrational modes, confirms the presence of IOMNPs in the synthesized nanosystem.

#### 2.1.2. Electron Microscopy Investigations

**Figure 2:**
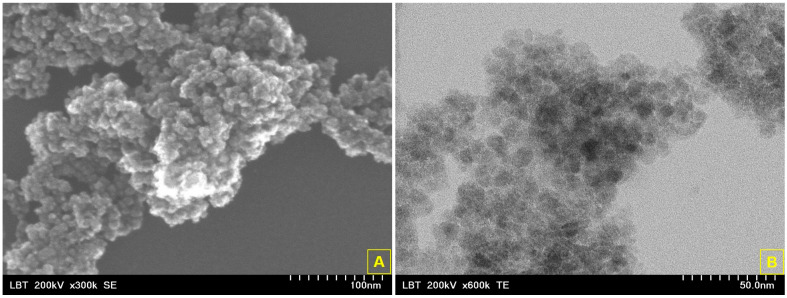
SEM (**A**) and TEM (**B**) images of the CHX-NPS nanosystem.

The size and shape of the synthesized CHX-carrier nanosystem based on maghemite NPSs (CHX-NPS) were investigated through Scanning Electron Microscopy—SEM—and Transmission Electron Microscopy—TEM. Figure 2 presents the microscopy images (SEM and TEM) of the CHX-NPS nanosystem at higher magnification for measurements (the first image at 300 kx) and for details (the second image at 600 kx). As regards the SEM image (Figure 2A), one can notice that the CHX-NPS nanosystem presents nanoparticles of nearly spherical shape, quasi-uniformly distributed. An aggregation of nanoparticles is observed, probably due to the large surface area to volume ratio.

By using ImageJ (https://imagej.nih.gov/ij/index.html, accessed on 25 July 2024), more than 100 randomly selected nanoparticles were measured, and the result revealed that the nanoparticles contained in the CHX-NPS nanosystem were in the nanometric domain (10 ± 1.8 nm) with the nearly spherical shape of 1.24 ± 0.3, determined from the TEM image (Figure 2B). Based on the mean size and standard deviation, determined from TEM micrographs using ImageJ software, version number 1.53f [https://imagej.nih.gov/ij/, accessed on 25 July 2024] one can determine the confidence intervals. To compute the confidence intervals, the following formula was used:

(1) $$Confidence\;Interval\;\left(CI\right)=\bar{X}\pm Z_{crit}\frac{\sigma }{\sqrt{n}}$$

where $\bar{X}$ is the sample mean, $Z_{crit}$ is the z-value corresponding to the desired confidence level from standard normal distribution (e.g., 1.96 for 95% confidence), σ is the sample standard deviation, and *n* is the sample size (100 NPs).

Therefore, the CI calculated was CI = {9.6472, 10.3528}, meaning that if we take any range of values from the data set, there will be a 95% probability that the value will lie between 9.6472 and 10.3528. Since we took into account a larger number of nanoparticles (more than 100 randomly selected nanoparticles), the margin of the error was smaller (10 ± 0.3528), and therefore the confidence interval was smaller, meaning a more precise estimate value.

#### 2.1.3. Energy-Dispersive Spectroscopy (EDS)

**Figure 3:**
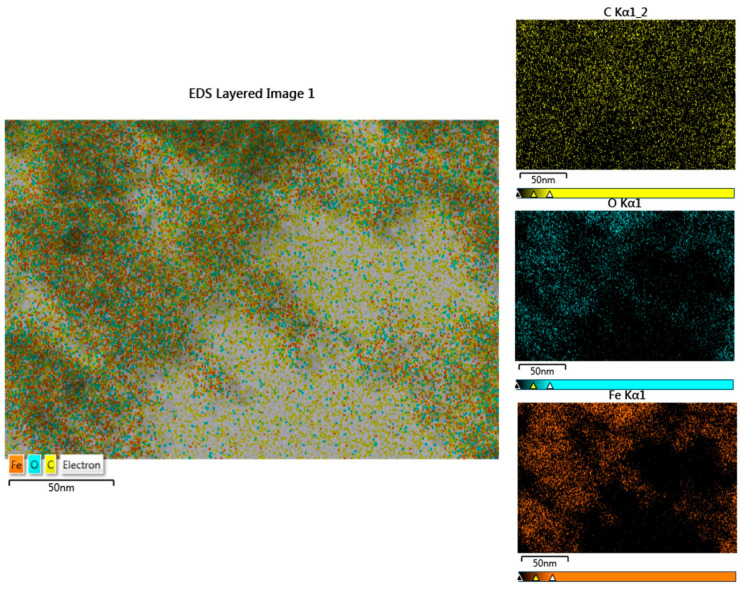
The EDS mapping of the CHX-NPS nanosystem.

Figure 3 shows the energy-dispersive spectroscopy (EDS) mapping of the CHX-NPS nanosystem, which was performed to determine the presence of dispersed and homogeneous elements contained in the sample. The EDS mapping for the CHX-NPS nanosystem shows the presence of Fe, O, and C atoms. The presence of a high amount of C atoms can be due to the carbon sputter coating of the CHX-NPS sample, applied for better conductivity, as well as due to the C atoms from the CHX structure.

#### 2.1.4. DLS Measurement of CHX-NPS Nanosystem

The dynamic light scattering method (DLS) was employed to assess maghemite nanoparticle size from the aqueous solution of the CHX-NPS nanosystem. Through DLS, we performed measurements regarding the values of the hydrodynamic diameter (Hd), polydispersity index (PDI), and zeta potential (ζ-potential) at 25 °C. The measurements revealed that the maghemite nanoparticles have a Hd = 79.65 nm, a PDI of 0.210, and a ζ-potential of −25.3 mV. The values obtained revealed that the nanoparticles have a narrow size distribution, being monomodal in nature (with a single population of nanoparticles). The value of the ζ-potential indicated high stability of the nanosystem against aggregation, an affirmation which is in agreement with the value obtained from the polydispersity index (PDI).

#### 2.1.5. The Loading and Release Capacity of CHX

The CHX loading capacity (%) of CHX onto maghemite nanoparticles was calculated to be 24.11%, and the release capacity (%) of CHX from the CHX-NPS nanosystem was 12.09% after 4 h of the experiment. Our findings demonstrate that a significant amount of CHX was loaded onto maghemite NPSs by the adsorption process. As regards the release capacity, zero-order kinetics was used to describe the release capacity, where CHX is released at a constant rate over time.

### 2.2. In Vitro Toxicological Screening

#### 2.2.1. Antibacterial Assay

The antibacterial activity of maghemite NPSs, the CHX-NPS nanosystem, and the CHX solution was assessed against Staphylococcus aureus (a Gram-positive bacterial strain), as well as against *Escherichia coli* and *Pseudomonas aeruginosa* (two Gram-negative bacterial strains), using various concentrations of all the tested samples (10, 50, and 100 μg/mL), and the results are presented in Table 1. By micro-dilution test, assessing the minimum inhibitory concentration (MIC—μg/mL) and minimum bactericidal concentration (MBC—μg/mL) determinations, the antimicrobial effect of the three test samples was measured. The results showed that all the samples tested present antibacterial activity against *S. aureus*, *E. coli*, and *P. aeruginosa* in a dose-dependent manner.

One can observe that maghemite NPSs present the highest inhibition on *E. coli* bacterium as compared to *P. aeruginosa* and *S. aureus*, but only at the high concentration tested (100 μg/mL). At the lowest concentration tested (10 μg/mL), the maghemite NPSs showed insignificant bacteriostatic and bactericidal activities on all three bacilli strains used, since the value recorded for the MIC and MBC exceeds 800 μg/mL.

The synthesized CHX-NPS nanosystem and the CHX solution showed the highest inhibition on *E. coli* followed by *S. aureus* and *P. aeruginosa*, respectively, when they were tested at the highest concentration (100 μg/mL). The attachment (functionalization) of CHX solution to the maghemite NPS surface slightly enhances the bacteriostatic and bactericidal activity on all three bacilli strains as the sample’s concentrations increase compared to the CHX solution tested alone.

#### 2.2.2. Cell Viability Evaluation

As a further step, to determine the safety use of the CHX-carrier nanosystem based on maghemite NPSs, an in vitro analysis was performed, to observe the impact of maghemite NPSs, the CHX-NPS nanosystem, and the CHX solution on the viability of two healthy cell lines of different origins (JB6 Cl 41-5a—epidermal murine cells—and HaCaT—human immortalized keratinocytes). Using the MTT assay (3-(4,5-dimethylthiazol-2-yl)-2,5-diphenyltetrazolium bromide), cell viability was determined at 24 h after treatment with maghemite NPSs, the CHX-NPS nanosystem, and the CHX solution (10, 50, 100 μg/mL) following a 1 and 2 min exposure.

**Figure 4:**
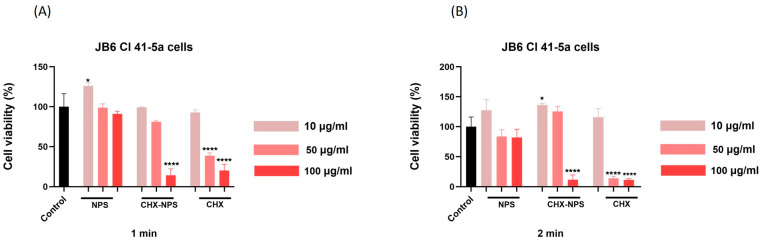
Graphical representation of the JB6 Cl 41-5a cells’ viability percentages at 24 h after exposure for 1 min (**A**) and 2 min (**B**) to 10, 50, and 100 μg/mL of maghemite NPSs, CHX-NPS nanosystem, and CHX solution. The results are presented as percentages (%) relative to untreated cells (control). All data are presented as mean ± SD values based on three independent experiments. To evaluate the statistical differences between the control untreated cells and the cells treated with the samples of interest, the one-way ANOVA test was performed followed by Dunnet’s post hoc multiple comparisons. ‘*’ indicates statistical significance (* *p* < 0.05 and **** *p* ≤ 0.0001).

In the case of JB6 Cl 41-5a murine cells, as shown in Figure 4 and Table 2, the test samples (NPSs, CHX-NPS, and CHX) induced a dose- and exposure time-dependent cytotoxic effect. The highest reduction in cell viability was calculated for the CHX solution after 2 min exposure (approximately 10%), whereas the smallest decrease in cells’ viability percentage was noted in the case of maghemite NPSs, where at the lowest concentration, cells appear to be stimulated by the application of this treatment.

Regarding the impact of the synthesized CHX-NPS nanosystem on JB6 Cl 41-5a cells, only the highest concentration tested (100 µg/mL) determined a significant reduction in cells’ viability, but at a lower extent than that of the CHX solution. At 1 min exposure to the medium concentration of 50 μg/mL, the synthesized CHX-NPS nanosystem reduces cell viability up to 81%, while the CHX solution decreases viability up to 38%.

**Figure 5:**
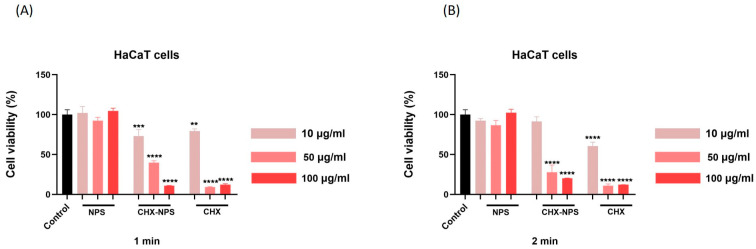
Graphical representation of the HaCaT cells’ viability percentages at 24 h after exposure for 1 min (**A**) and 2 min (**B**) to 10, 50, and 100 μg/mL of maghemite NPSs, CHX-NPS nanosystem, and CHX solution. The results are presented as percentages (%) relative to untreated cells (control). All data are presented as mean ± SD values based on three independent experiments. The one-way ANOVA test was performed to evaluate the statistical differences between the control untreated cells and the cells treated with the samples of interest, followed by Dunnet’s post hoc multiple comparisons. ‘*’ indicates statistical significance (** *p* < 0.01; *** *p* < 0.001; and **** *p* ≤ 0.0001).

Similarly, in the case of HaCaT cells (Figure 5, Table 3), the lowest viability percentages were noticed for CHX solution treatment with a significant decrease in cell viability starting at a dose of 50 μg/mL for both exposure periods. Maghemite NPSs did not cause a marked decrease in cell viability (the lowest value being 86%). The synthesized CHX-NPS nanosystem reduced cell viability in a concentration-dependent manner, with the greatest cell impairment being observed at the highest concentration (15% at 100 μg/mL).

#### 2.2.3. Hoechst 33342 Nuclear Staining

**Figure 6:**
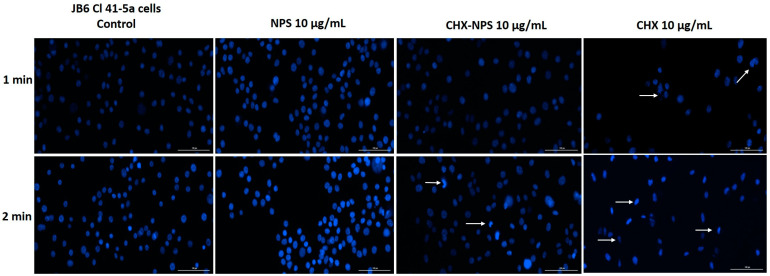
Representative images of cell nuclei in JB6 Cl 41-5a cells at 24 h after exposure for 1 and 2 min to 10 μg/mL concentration of maghemite NPSs, CHX-NPS nanosystem, and CHX solution. The white arrows represent massive condensed nuclei with a reduction in size, and frequent dysmorphologies. Images were captured at 20× magnification. The scale bar indicates 100 μm.

Hoechst 33342 nuclear staining was employed to analyze the potential cell nuclei changes at 24 h after exposure to the samples of interest for 1 min and 2 min. To perform this analysis, each sample’s lowest (10 μg/mL) and highest (100 μg/mL) concentrations were selected. The lowest concentration of test samples—10 μg/mL—determined in JB6 Cl 41-5a cells (Figure 6) demonstrated the following: (i) no noticeable changes were detected in cell nuclei after treatment with maghemite NPSs at any of the exposure periods; (ii) dysmorphologies and condensation of nuclei can be observed at 2 min after exposure to the synthesized CHX-NPS nanosystem, but to a lesser extent than in the case of the CHX solution; and (iii) numerous dysmorphologies even at 1 min cell’s exposure to the CHX solution and after 2 min cell’s exposure, massive condensed nuclei with a reduction in size, and frequent dysmorphologies were detected (the changes are marked through the white arrows).

**Figure 7:**
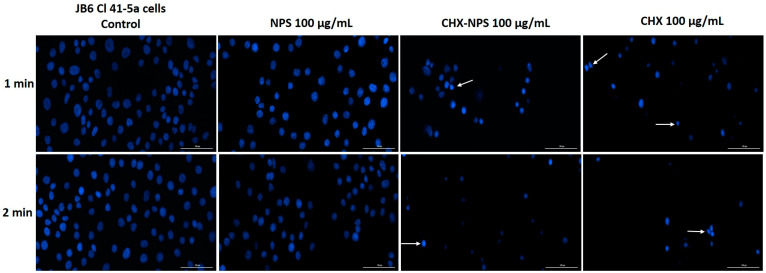
Representative images of cell nuclei in JB6 Cl 41-5a cells at 24 h after exposure for 1 and 2 min to 100 μg/mL concentration of maghemite NPSs, CHX-NPS nanosystem, and CHX solution. The white arrows represent nuclei damaged (condensed and significantly size reduced, as well as dysmorphologies). Images were captured at 20× magnification. The scale bar indicates 100 μm.

Exposure for 1 and 2 min to the highest concentration of the samples—100 µg/mL—both synthesized the CHX-NPS nanosystem, and the CHX solution led to damaged nuclei in JB6 Cl 105 41-5a cells (Figure 7). Under CHX solution treatment, the nuclei were significantly reduced in size and condensed at both exposure times, whereas for the synthesized CHX-NPS nanosystem, nuclei with dysmorphologies and reduced in size could be observed predominantly at the 2 min exposure period. The maghemite NPSs did not cause substantial nuclear changes even at the highest concentration compared to those of the control.

**Figure 8:**
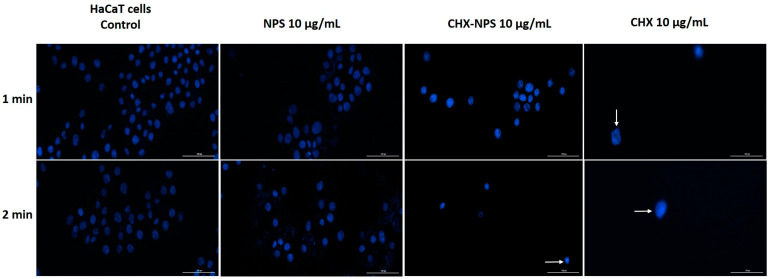
Representative images of cell nuclei in HaCaT cells at 24 h after exposure for 1 and 2 min to 10 μg/mL concentration of maghemite NPSs, CHX-NPS nanosystem, and CHX solution. The white arrows represent condensed and shape-changed nuclei. Images were captured at 20× magnification, the scale bar indicates 100 μm.

In HaCaT cells, maghemite NPSs exhibited a similar effect as in the case of murine cells (JB6 Cl 41-5a), with no alterations in nuclei shape as compared to control cells. As can be seen in Figure 8, the lowest concentration of CHX solution of 10 μg/mL, produced condensed and shape-changed nuclei at both exposure times (the changes are marked through white arrows). The synthesized CHX-NPS nanosystem, at the same concentration (10 μg/mL), caused the greatest alteration of nuclei following the 2 min exposure period, characterized by small, rounded, and condensed nuclei.

**Figure 9:**
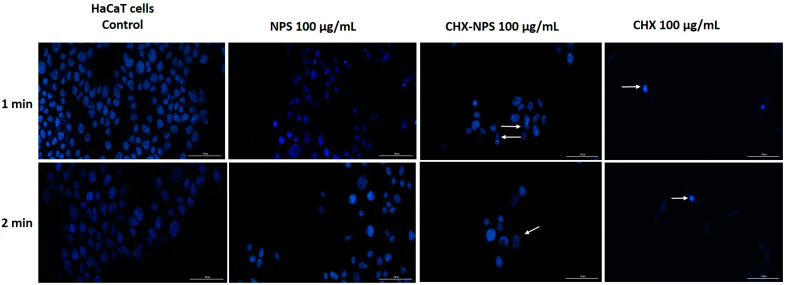
Representative images of cell nuclei in HaCaT cells at 24 h after exposure for 1 and 2 min to 100 μg/mL maghemite NPSs, synthesized CHX-NPS nanosystem, and CHX solution. The white arrows represent frequent dysmorphologies and condensation of nuclei. Images were captured at 20× magnification. The scale bar indicates 100 μm.

Furthermore, when tested with the highest concentration (100 μg/mL) of maghemite NPSs, the CHX-NPS nanosystem, and the CHX solution, on HaCaT cells, the CHX solution caused increased dysmorphologies compared to those of the other samples tested. At both exposure times, the CHX solution caused shrinkage of nuclei, rounding, and condensation (Figure 9). In the case of the synthesized CHX-NPS nanosystem, frequent dysmorphologies and condensation of nuclei occurred. After maghemite NPS treatment, there were no substantial changes in the shape of the nuclei.

#### 2.2.4. Apoptotic Index

**Figure 10:**
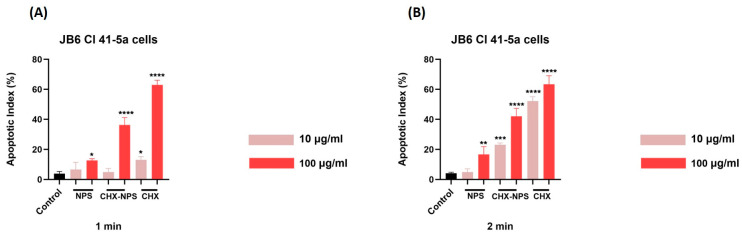
Graphical representation of apoptotic index analysis for JB6 Cl 41-5a cells 24 h after treatment with maghemite NPSs, CHX-NPS nanosystem, and CHX solution at 1 (**A**) and 2 (**B**) minutes of exposure in concentrations of 10 and 100 μg/mL. Data are presented as the apoptotic index (%) reported for control (untreated cells). The results are presented as percentages (%) relative to untreated cells (control). All data are presented as mean ± SD values based on three independent experiments. The one-way ANOVA test was performed to evaluate the statistical differences between the control untreated cells and the cells treated with the samples of interest, followed by Dunnet’s post hoc multiple comparisons. ‘*’ indicates statistical significance (* *p* < 0.05; ** *p* < 0.01; *** *p* < 0.001; and **** *p* ≤ 0.0001).

According to the formula for the calculation of the apoptotic index, the pro-apoptotic effect seems to be dependent on the concentration used. For JB6 Cl 41-5a cells—Figure 10 and Table 4—the results indicate that the highest pro-apoptotic effect is represented by the CHX solution at both exposure periods. According to the results, the concentration of 100 μg/mL induces the highest pro-apoptotic effects for either sample.

**Figure 11:**
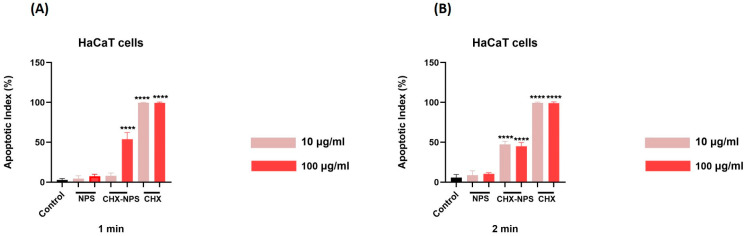
Graphical representation of apoptotic index analysis for HaCaT cells 24 h after treatment with maghemite NPSs, CHX-NPS nanosystem, and CHX solution at 1 (**A**) and 2 (**B**) minutes of exposure in concentrations of 10 and 100 μg/mL. Data are presented as the apoptotic index (%) reported for control (untreated cells). The results are presented as percentages (%) relative to untreated cells (control). All data are presented as mean ± SD values based on three independent experiments. The one-way ANOVA test was performed to evaluate the statistical differences between the control untreated cells and the cells treated with the samples of interest, followed by Dunnet’s post hoc multiple comparisons. ‘*’ indicates statistical significance (**** *p* ≤ 0.0001).

In the same manner, for HaCaT cells (Figure 11, Table 5), one can observe that the CHX solution produces the highest pro-apoptotic effects at both exposure times (1 and 2 min). For the CHX-NPS nanosystem, the 2 min exposure period causes for both concentrations tested an increase in the apoptosis index.

### 2.3. In Ovo Toxicological Screening

**Figure 12:**
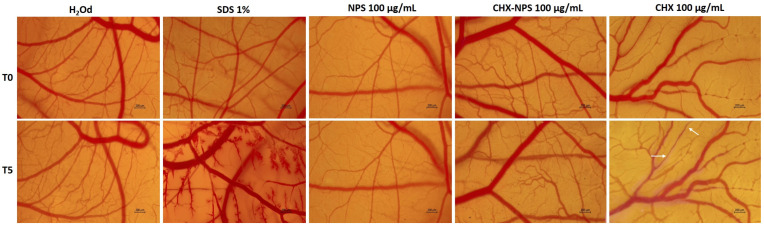
Representative images of the appearance of the chorioallantoic membrane vessels at time T0 (before sample application) and at time T5 (5 min after sample application). H_2_Od was used as a negative control and SDS 1% as a positive control. The white arrows represent the changes made by the CHX solution, meaning signs of lysis, coagulation, and slight hemorrhage. The scale bar indicates 200 µm.

To observe comparatively the irritant effect produced by maghemite NPSs, the CHX-NPS nanosystem, and the CHX solution at 100 μg/mL, the HET-CAM test was performed based on which the irritation score was calculated (Figure 12, Table 6). Distilled water (H_2_Od) was used as a negative control, while SDS (sodium dodecyl sulfate) 1% was used as a positive control. Thus, according to the results, H_2_Od did not cause any sign of irritation on the chorioallantoic membrane vasculature and was classified as a non-irritant substance. SDS 1% was caused in a short period of hemorrhage, lysis, and coagulation and thus was classified as a severe irritant substance. Maghemite NPSs and CHX-NPS nanosystems were classified as non-irritating substances because no signs of irritation appeared during the 5 min. The CHX solution, however, induced signs of lysis, coagulation, and slight hemorrhage and was classified as an irritating substance. In addition, a thinning of the vessels can be observed in several places in the case of the CHX solution treatment (the changes observed are marked through the white arrows—Figure 12).

## 3. Discussion

Chlorhexidine, a bisbiguanide compound, is widely used in both dentistry and medicine due to its strong antimicrobial properties. In dentistry, chlorhexidine is predominantly used as a mouthwash or gel to combat oral pathogens. A mouthwash with 0.2% chlorhexidine effectively reduces the microbial load in the oral cavity, thereby reducing the risk of conditions such as gingivitis and periodontitis [59,60]. The mechanism of action involves disruption of bacterial cell membranes, leading to cell lysis and death, which is particularly effective against oral biofilms that contribute to dental caries and periodontal disease [61,62]. However, the use of CHX is not without disadvantages. Long-term use can lead to adverse effects, including extrinsic tooth staining, altered taste sensation, and mucosal irritation [63]. These side effects have led to recommendations for short-term use, particularly after surgical procedures or during the acute phases of oral disease [49,50,64]. Moreover, the toxicity of CHX at higher concentrations raises concerns regarding its safety and efficacy in clinical settings. Chronic toxicity arises from exposure over a long period of time to a particular agent, and under this aspect it can progress to decreased quality of life, impact on the psyche, behavioral changes, or even death [65]. Moreover, a delay in the healing process of the skin may form a chronic wound that can be the point of bacterial colonization [66], especially in the oral environment where there is continuous moisture and enclosed space. For this reason, the present study aimed to develop a formulation that would present lower toxicity and increased antibacterial activity, confirmed by in vitro and in ovo investigations. In addition to this aspect, the effectiveness of chlorhexidine may be limited by its solubility and stability in different environments, which requires the development of advanced transport systems that can enhance its bioavailability and therapeutic outcomes [67,68].

Solid nanosystems have gained attraction due to their ability to improve the pharmacokinetics and pharmacodynamics of therapeutic agents. Furthermore, the interaction of chlorhexidine with IOMNPs can enhance its antimicrobial properties through mechanisms such as increased surface area for interaction with microbial cells and the ability to generate reactive oxygen species upon exposure to light, further augmenting its antimicrobial effects [5]. The incorporation of chlorhexidine into nanosystems not only improves its stability and release characteristics but allows for targeted delivery to infected sites, which is crucial in managing localized infections effectively [67,68].

In the above context, the present study reports the synthesis of a nanoscale system for CHX delivery using IONPs (maghemite) with an improved toxicological profile as compared to that of the CHX solution, motivated by the wide use of CHX in the medical fields (dentistry, surgery) and, in particular, by their known side-effects [4,21,22,69,70]. Therefore, the alternative strategy to avoid CHX side effects proposed in the present study was incorporating CHX 2% solution into maghemite nanoparticles. The choice of maghemite among the other iron oxides was based on the following considerations: high availability, biocompatibility, increased stability, large surface area in certain conditions [71], environmental sustainability, magnetic properties, superparamagnetic behavior, and absence/very low toxicity [28,72,73]. For the incorporation of CHX, γ-Fe_2_O_3_ nanopowder obtained via combustion synthesis was used followed by H_2_O_2_ treatment, as Ianoş and co-workers described in detail, regarding the synthesis process [71]. Following treatment with H_2_O_2_, the physicochemical properties of maghemite were improved (a decrease in carbon content, a significant increase in the surface specific area, and an improvement in magnetic properties). In addition, it seems that treatment with H_2_O_2_ ensures the sterilization of γ-Fe_2_O_3_ nanoparticles, which is a significant requirement in biomedical applications.

After we synthesized the CHX-NPS nanosystem, the attachment of disinfectant on the maghemite nanoparticles’ surface was confirmed as well as the nanometric scale. FTIR analysis confirmed the attachment of 2% CHX solution onto IONPs by absorption bands located at 3246.20/cm, 2164.13/cm, 1654.92/cm, 1151.50/cm, 889.18/cm, and 520.78/cm. Our results agree with those of research studies reported in the literature [5,21]. Moreover, it can be noted that the main absorption bands of IONPs remained in the FTIR spectrum, after CHX solution incorporation. The band strengthens the inorganic domain characteristic for the Fe-O stretching vibration from iron oxide magnetic nanoparticles. According to the literature, the peaks recorded under 600/cm can be assigned to the Fe-O vibration confirming the presence of iron oxide magnetic nanoparticles [74,75,76]. The results revealed that the 2% CHX solution was incorporated into nearly spherical IONPs with a narrow size diameter of 10 ± 2.8 nm, in which Fe, O, and C atoms were quasi-uniformly distributed, according to EDS mapping [5,21]. This investigation is well correlated also with DLS measurements, which revealed a hydrodynamic diameter of 79.65 nm and a zeta potential of −25.3 mV confirming the high stability of the nanoparticles. Chlorhexidine (CHX) absorption on the surface of magnetic nanoparticles is a critical area of research, particularly in drug delivery systems and antimicrobial applications. Due to their unique properties, the controlled release of chlorhexidine is allowed. Our results showed that a significant amount of CHX was loaded onto the maghemite nanoparticles (24.11%), and a release capacity of 12.09% after 4 h of observation, highlighting the use of magnetic nanoparticles as a solid matrix. Our findings align with the findings of Cai et al. [77], who stated that encapsulation not only enhances the stability of CHX but also allows for its gradual release, which is beneficial for maintaining therapeutic concentrations over time. In addition, the work developed by Barbour et al. [78] illustrates the development of novel antimicrobial nanoparticles that can serve as slow-release devices for CHX, enhancing its antimicrobial properties while minimizing the risk of resistance. This observation is in agreement with the findings of Ebrahim et al. [79], who noted that the magnetic properties of nanoparticles can be harnessed for targeted delivery, thereby improving the therapeutic outcomes of CHX. To calculate the release capacity of chlorhexidine from maghemite NPSs, it is essential to consider various factors including the formulation of the nanoparticles, the release medium, and the methodology employed for quantifying the released CHX. Firstly, the formulation of the nanoparticles significantly influences the release capacity. For instance, CHX hexametaphosphate nanoparticles (CHX-HMP NPs) have been shown to provide the sustained release of CHX when subjected to an aqueous environment, highlighting their potential as effective drug delivery systems [80]. Additionally, the use of mesoporous nanoparticles has been reported to facilitate the controlled release of CHX due to their porous structure, which allows for the entrapment and gradual release of the drug [81,82]. The release medium also plays a crucial role in determining the release kinetics which can be evaluated by dispersing drug-loaded nanoparticles in different buffer solutions and measuring the concentration of CHX at specified intervals [83]. Moreover, the kinetics of CHX release can be modeled using various mathematical equations as well as techniques to accurately measure the concentration of CHX in the release medium, allowing for a precise calculation of the release capacity [84,85].

It is well known that CHX is an antiseptic agent widely used in oral health. In addition to its use in oral health, CHX has found applications in medicine, particularly in the prevention of healthcare-associated infections, due to its strong antimicrobial properties. Its use in preoperative skin antisepsis has been shown to significantly reduce the incidence of surgical site infections [60]. The effectiveness of chlorhexidine in these scenarios highlights its importance as a broad-spectrum antimicrobial agent in clinical practice. Therefore, in the present study, the antimicrobial investigation of the interest samples (maghemite NPSs, CHX-NPS nanosystem, and CHX solution) was conducted against three microorganisms: a Gram-positive bacterium—*Staphylococcus aureus*—and two Gram-negative bacteria—*Escherichia coli* and *Pseudomonas aeruginosa*. This carried out through the disc-diffusion assay. We chose to select these types of bacilli strains since they are involved in skin pathology and, in the present study, this was followed by initially demonstrating the in vitro safety profile of the synthesized CHX-NPS nanosystem, on healthy human keratinocytes as well as on healthy murine epidermal cell lines, before being used in oral pathology. Moreover, it was stated that *Staphylococcus aureus*, *Escherichia coli*, and *Pseudomonas aeruginosa* are pathogens that are frequently isolated from infections related to the surfaces of biomaterial implants [86]. This is why in the present study these three bacilli strains were selected. Our outcomes have shown that all the samples tested present antibacterial activity against the Gram-positive and Gram-negative bacilli strains in a dose-dependent manner. Maghemite NPSs were more active against *E. coli* bacterium, but only at the highest concentration tested (100 μg/mL) requiring an MIC value of 80 μg/mL and an MBC value of 100 μg/mL, while at the lowest concentration tested (10 μg/mL), maghemite NPSs almost did not show antibacterial activity. Therefore, the *E. coli* bacterium needs quite high maghemite NPS concentrations to inhibit its growth. On the contrary, when the CHX solution was attached to the surface of maghemite NPSs, the CHX-NPS nanosystem showed significant antimicrobial activity even better than the activity of the CHX solution tested alone. A significant decrease was observed in the MIC and MBC, with an increase in the concentration of the nanosystem. Our findings are similar to those reported in the literature [47,48,50,87]. A concentration-dependent antimicrobial activity of IONPs on different bacilli strains was reported also by other research groups [88,89,90,91]. Therefore, it was shown that the incorporation of chlorhexidine (CHX) into IONMPs represents a promising strategy to enhance antimicrobial activity, particularly against Gram-negative bacteria such as *Escherichia coli* and *Pseudomonas aeruginosa*. One of the primary mechanisms by which CHX-embedded IONMPs enhance antimicrobial activity is through improved cellular uptake. The surface properties of nanoparticles, including charge and size, significantly influence their interaction with bacterial membranes. Positively charged nanoparticles tend to have better adhesion to negatively charged bacterial surfaces, facilitating uptake [92]. This is particularly relevant for Gram-negative bacteria, which possess an outer membrane that can act as a barrier to many antimicrobial agents. The presence of IONMPs can disrupt this barrier, allowing for increased penetration of CHX into the bacterial cell [93]. Additionally, the magnetic properties of these nanoparticles enable targeted delivery through external magnetic fields, further enhancing their uptake in specific areas of infection [94]. Prolonged release of CHX from IONMPs is another critical factor contributing to their enhanced antimicrobial efficacy. The controlled release mechanism can be achieved through various formulations that allow for sustained release of the drug over time, maintaining effective concentrations at the site of infection [95]. This is particularly significant for combating resistant strains of bacteria, as prolonged exposure to antimicrobial agents can increase the likelihood of bacterial cell death and reduce the chances of resistance development [96]. As regards the significant effect of the nanosystem on the Gram-negative bacteria (*Escherichia coli* and *Pseudomonas aeruginosa*), the unique mechanism of action offered by this type of nanosystem may bypass the traditional resistance pathways of these types of bacteria, offering a new approach to managing infections caused by these resistant organisms [97]. It is well known that these pathogens are notorious for their resistance to multiple classes of antibiotics, making infections difficult to treat [98,99]. The ability of CHX-embedded IONMPs to effectively target and kill these bacteria not only addresses immediate infection concerns but also contributes to broader public health efforts to combat antibiotic resistance [100].

As regards the enhanced killing effect of the CHX-NPS nanosystem compared to the effect of the CHX solution, once again our study is in agreement with the study reported by Tokajuk and co-workers [5], who reported that magnetic nanoparticles functionalized with CHX could significantly enhance its bactericidal and fungicidal activities against both planktonic and biofilm-forming microorganisms compared to free chlorhexidine. This is particularly relevant in the context of biofilm-associated infections, where traditional antibiotic therapies often fail due to the protective nature of biofilms [5,101]. In vitro studies have demonstrated that CHX exhibits rapid antimicrobial activity, effectively reducing the viability of various microorganisms within minutes of exposure [102]. For instance, CHX has been shown to eliminate aerobic microorganisms such as *Staphylococcus aureus* and *Candida albicans* in less than 30 s [102]. The ability of CHX to inhibit the growth of opportunistic pathogens, such as *Candida albicans*, further supports its use also in immunocompromised patients [103]. This antifungal activity is particularly relevant in the context of oral candidiasis, where CHX has been shown to inhibit the adhesion of *Candida* to mucosal surfaces, thereby preventing infection [104]. Therefore, in addition to enhancing the antimicrobial efficacy of CHX, the development of these nanosystems can also address challenges related to microbial resistance. Comparatively, other nanosystems documented in the literature have shown promise in terms of antimicrobial efficacy but may also present challenges [105]. However, they can also exhibit significant cytotoxicity, which raises concerns regarding their safety profile [106,107]. In contrast, while chlorhexidine has established cytotoxic effects, its combination with nanoparticles has been shown to enhance its antibacterial properties while potentially reducing cytotoxicity [108,109]. Similarly, the encapsulation of CHX in mesoporous silica nanoparticles has been shown to facilitate controlled release and improve bioavailability, which can lead to more effective treatment outcomes against oral biofilms [110]. Therefore, the enhanced antimicrobial efficacy, potential reduction in side effects, and lower risk of resistance development associated with nanosystem formulations make them a promising avenue for improving the therapeutic application of CHX.

The safety and biocompatibility of IOMNPs are critical factors that influence their clinical application. Their in vitro toxicity remains a critical concern that necessitates thorough investigation. The cytotoxic effects of IOMNPs can vary significantly based on several factors, including their size, surface coating, and the specific cell lines used for testing [111,112]. The size and surface characteristics of IOMNPs play a pivotal role in determining their cytotoxic effects. Smaller nanoparticles tend to exhibit higher cellular uptake and, consequently, greater toxicity [113]. Furthermore, the surface coating of IOMNPs significantly influences their biocompatibility [113,114,115]. It has been stated that IOMNPs could degrade into free iron ions in acidic environments, which can further exacerbate oxidative stress through Fenton-type reactions [116]. This degradation contributes to cytotoxicity and raises concerns regarding iron accumulation in tissues, which can lead to long-term health implications [117]. Moreover, the cellular uptake of IOMNPs can disrupt normal cellular signaling pathways, leading to inflammation and cell death [118]. Besides all this, the time and dose dependency of IOMNP toxicity is another crucial aspect to consider. The importance of optimizing dosage and exposure duration in therapeutic applications has been shown to mitigate the potential cytotoxic effects of IOMNPs [119]. Additionally, the concentration of IOMNPs used in experiments can significantly influence the observed toxicity; higher concentrations are generally associated with increased cytotoxicity [120].

In the context of the above, an in vitro toxicological screening was performed regarding the biological effect of maghemite NPSs, the synthesized CHX-NPS nanosystem, as well as the CHX solution. The treatment regimen on the cell lines was chosen to mimic the clinical application of CHX to patients. Thus, cells were treated for 1 and, respectively, 2 min with CHX, then washed with PBS to remove excess, and incubated for 24 h. All experiments were performed after the 24-hour incubation period. This mode of treatment is based on the premise that the most popular applications of CHX are as a disinfectant and antiseptic, and in dental applications it is very commonly found in mouthwashes [121]. However, one aspect to emphasize is that CHX is absorbed on different surfaces in the oral cavity (mucosa or pellicle-coated teeth), therefore performing a sustained antimicrobial activity, which restricts bacterial growth for about 24 h; thus, CHX is considered to continue its action even after effective removal from tissue or mucosa. Having all this in view, the treatment of cells was intended to resemble conventional treatments of tissues or mucous membranes. Our findings concerning maghemite NPSs indicate very low toxicity in JB6 Cl 41-5a murine cells (Figure 4) and HaCaT human keratinocytes (Figure 5), even at the highest concentration tested—100 µg/mL.

As concerns CHX in vitro toxicity, its cytotoxic effects have raised concerns regarding its safety profile, particularly when used in higher concentrations. Numerous studies have investigated the in vitro toxicity of chlorhexidine on various cell types [1,70,122,123,124,125,126], revealing significant cytotoxicity at concentrations commonly used in clinical practice. In our experimental design, using JB6 Cl 41-5a murine epidermal cells and HaCaT human immortalized keratinocytes, the CHX solution proved a dose- and time-dependent cytotoxic effect characterized by a significant decrease in cells’ viability and apoptotic-like nuclear changes (Figure 4, Figure 5, Figure 6, Figure 7, Figure 8 and Figure 9), data that are in agreement with the previously stated results. Since most of the studies that evaluated CHX toxicity used in vitro model cells of oral and bone origin, and CHX is also used as a disinfectant for skin application, we selected in vitro model cells of skin origin: JB6 Cl 41-5a murine epidermal cells and HaCaT human keratinocytes. Both cell lines selected for investigation represent useful experimental models in correlating the multiple clinical uses of CHX. Keratinocytes were chosen because they predominate in the layered squamous epithelium of the gingiva and have also been studied in this regard. HaCaT cells have the advantage that they are similar in the presence of the main surface markers, functionality, and morphology to isolated keratinocytes. The JB6 Cl 41-5a cells are epidermal cells, which in the same manner were selected as a model for the evaluation of cutaneous safety. By choosing these two distinct types of cell lines, it was intended to achieve the widest possible applicability of the compound under investigation [121].

In vitro studies have demonstrated that CHX can inhibit the adhesion and growth of various cell types, including keratinocytes and fibroblasts. For instance, it has been shown that concentrations above 0.002% of chlorhexidine gluconate induce toxicity in keratinocytes and fibroblasts, suggesting that even low concentrations can have detrimental effects on cellular viability [127]. The outcomes of this study align with the findings from Sedaghat and co-workers [128], who noted that CHX exhibits toxic effects on keratinocytes and fibroblasts at concentrations as low as 0.05%. Studies have also shown that human gingival fibroblasts and periodontal ligament cells are adversely affected by exposure to CHX [126]. In addition, CHX can induce apoptosis in cell lines of L929 mouse fibroblasts, which may reflect similar effects in human cells [129]. The mechanism of chlorhexidine’s cytotoxicity appears to involve damage to cellular structures and functions. Research indicates that CHX disrupts cell membranes, leading to leakage of intracellular components, which is a common pathway for cytotoxic agents [130]. This is particularly concerning in the context of dental procedures where chlorhexidine is often used as an irrigate or disinfectant.

Although the cytotoxic effects of CHX on human cells, particularly fibroblasts, have raised concerns about its safety profile [66,131], the incorporation of CHX into nanosystems may mitigate these cytotoxic effects by allowing controlled release and localized administration, thereby reducing systemic exposure and increasing safety [66,77]. The formulation of CHX-loaded nanosystems has been explored in various studies, highlighting the importance of optimizing the carrier matrix to achieve desired release profiles and antimicrobial efficacy [68,132]. In vitro studies have demonstrated that CHX-loaded IOMNPs exhibit enhanced antimicrobial activity compared to that of free CHX. This is attributed to the improved retention and uptake of the drug by microbial cells, facilitated by the nanoparticles’ unique properties [133]. The use of IOMNPs not only enhances the solubility of CHX but also allows for controlled release, which can prolong its therapeutic effects. It was reported that the underlying cytotoxic mechanism of NPs is ROS formation and mitochondrial disruption [134].

In the present study, the incorporation of CHX solution onto the maghemite NPs surface was reported and led to obtaining the CHX-NPS nanosystem with a lower cytotoxic effect as compared to that of the CHX solution (Figure 4 and Figure 5). HaCaT human keratinocytes proved to be more sensitive to the CHX-NPS nanosystem as JB6 Cl 41-5a murine epidermal cells, with a reduction in cells’ viability and nuclear changes being observed starting with the concentration of 50 µg/mL (Figure 4, Figure 5, Figure 6, Figure 7, Figure 8 and Figure 9), but at a lower extent relative to the CHX solution, whereas at the highest concentration tested, the CHX-NPS nanosystem and CHX solution exhibited similar cytotoxic behavior. Similar results, meaning reduced toxicity at low concentrations and comparable cytotoxic effects at high concentrations, were described by Araujo and co-workers [22], as well as by Tokajuk and co-workers [5]. According to the literature and the results of the current study, the encapsulation of CHX in nanoparticles confers potential benefits in terms of dose optimization, decreased in vitro toxicity, and improved antimicrobial effect. In clinical practice, the use of nanocomplexes could lead to a decrease in the side effects of CHX, but also to a higher therapeutic effect at optimized doses.

The in ovo toxicological screening showed that the maghemite NPSs as well as the newly synthesized CHX-NPS nanosystem belong to the category of non-irritating substances because they did not produce signs of lysis, coagulation, or hemorrhage on the chorioallantoic membrane of the embryonated hen’s egg. The chorioallantoic membrane is highly vascularized and is generally used for nourishment or gas exchange. It consists of three layers (namely the chorionic epithelium, mesenchymal epithelium, and allantoic epithelium) each of which serves its role [135]. Because CHX comes into direct contact with oral mucosa as well as skin tissues during its clinical use, in ovo experiments, especially the HET-CAM test, are recognized for their applicability to observe the irritant effects on mucous membranes and for their correlation with the results indicated by dermal irritation tests [136,137]. Therefore, HET-CAM constitutes a versatile technique that was previously used for biocompatibility or safety purposes for numerous agents or materials [138].

Overall, the multifunctional nature of IOMNPs allows for the simultaneous delivery of chlorhexidine and other therapeutic agents, potentially leading to synergistic effects. By combining chlorhexidine with other antimicrobial agents, one can enhance overall efficacy while reducing the likelihood of resistance development [139]. This approach is particularly relevant in the context of treating multidrug-resistant infections, where traditional therapies may fail. The ability to tailor the composition and surface characteristics of IOMNPs also enables the development of personalized medicine strategies, where treatment can be optimized based on individual patient needs. Also, as a future research direction up to the clinical verification stage, studies should focus on the research of nanosystems in vivo to better observe the action of CHX-NPS in experimental models as close as possible to the human body.

## 4. Materials and Methods

### 4.1. Synthesis of the CHX-NPS Nanosystem

The carrier nano-matrix was composed of iron oxide magnetic nanoparticles (maghemite), obtained by the combustion method, according to the method described in detail by Ianos and co-workers [71]. Briefly, for obtaining iron oxide magnetic nanoparticles (IOMNPs), Fe(NO_3_)_3_·9H_2_O 96% (Carl Roth, Karlsruhe, Germany) as the oxidizing agent and C_6_H_18_N_4_ ≥ 97% (Honeywell Riedel-de Haën, Bucharest, Romania) as the reducing agent (fuel) were used, in a molar ratio of 1:1.3. The combustion reaction took place in a heating mantle, at 400 °C, after the oxidizing agent and the fuel were dissolved in 40 mL distilled water. To avoid the oxidation of magnetic nanoparticles, the evolving gases were bubbled in a beaker flask filled with distilled water. At the end of the combustion reaction between the iron nitrate and triethylenetetramine (after 30 min), a black-brown powder was obtained. Each gram of powder was treated with a 30% H_2_O_2_ solution (Silal Trading, Bucharest, Romania), and the mixture was subjected to mechanical stirring for 1 h. After evaporation and dryness, a reddish-brown powder was obtained. Some main characteristics of the obtained IOMNPs (maghemite) through the combustion method used as a nano-carrier for CHX solution delivery are presented in Table 7.

After H_2_O_2_ treatment, the BET surface area and saturation magnetization increased significantly to 192 m^2^/g.

The immobilization of 2% chlorhexidine (CHX) solution onto iron oxide magnetic nanoparticles was achieved by absorption of the chloride group from CHX into the maghemite NPSs’ surface. In total, 5 mg of maghemite nanoparticles was mixed with 2 mL of 2% chlorhexidine digluconate solution (Gluco Chex-In 2%) provided by CER—83 KAMED (Stalowa Wola, Polonia), and the mixture was continuously stirred at 250 rpm at a controlled temperature of 25 °C in an Environmental Shaker Incubator ES-20/60 BioSan (Riga, Latvia) for 4 h. Hereinafter, the precipitate was characterized and tested by conducting in vitro and in ovo studies.

### 4.2. Characterization of the CHX-NPS Nanosystem

The FTIR spectroscopy was performed by using the Prestige-21 spectrometer from Shimadzu Corporation (Duisburg, Germany) at room temperature. The 2% CHX solution as well as the newly obtained CHX-NPS nanosystem were analyzed to confirm the attachment of CHX onto the maghemite NPSs’ surface. To identify the functional groups in both samples, the FTIR spectra were obtained in the presence of KBr pellets. The analyses were performed at room temperature (22 ± 2 °C), in the spectral region ranging from 4000/cm to 400/cm with a resolution of 4/cm. The FTIR analysis was made according to the perfect match between the absorption bands recorded from both samples and the absorption bands frequency from the library [140].

The morphology and ultrastructure of the CHX-NPS nanosystem were assessed by scanning electron microscopy (SEM), using a Hitachi SU8230 cold field emission gun STEM microscope (Chiyoda, Tokyo, Japan), equipped with EDX detectors X-Max^N^ 80 from Oxford Instruments (Abingdon, UK). The analysis was carried out in a high-vacuum mode acceleration voltage of 30 kV, with secondary electron detectors at two magnification orders.

The particle size of the CHX-NPS nanosystem was evaluated by transmission electron microscopy (TEM), using a Hitachi HD2700 cold field emission gun STEM microscope (Chiyoda, Tokyo, Japan), equipped with two windowless EDX detectors X-Max^N^ 100 from Oxford Instruments (UK).

To prepare the sample for TEM investigation, a drop of the synthesized CHX-NPS nanosystem suspended in an aqueous solution (7 μL) was placed on a carbon-coated copper grid and dried at room temperature (22 ± 2 °C). After that, multiple micrographs were taken at 200 kV acceleration voltage. The synthesized CHX-NPS nanosystem was sputter-coated with 6 nm of carbon (Agar Automatic Sputter Coater, Essex, UK) for better conductivity and high-resolution imaging. In addition, the EDS mapping of the nanosystem was determined. By using ImageJ software, version number 1.53f (https://imagej.nih.gov/ij/, accessed on 25 July 2024), the CHX-NPS size and aspect ratio statistics were determined from TEM images.

To measure the hydrodynamic diameter (Hd), the polydispersity index (PDI), and the zeta potential, dynamic light scattering (DLS) was performed, using a Zetasizer Nano ZS (Malvern Instruments, Worcestershire, UK). The maghemite particle size was measured through photon correlation spectroscopy, ranging from 0.4 nm to 9 μm, at 25 °C. The zeta potential was measured using the electrophoretic light scattering method. The dispersant used was distilled water with a refractive index of 1.3329 and a viscosity of 0.8879 cP.

To determine chlorhexidine loading (%), the CHX-NPS sample was dissolved in hydrochloric acid, and the entire mixture was subjected to a vigorous stirring and centrifuged, and the collected supernatant was analyzed through high-performance liquid chromatography (HPLC), using a 6120 LC-MS analytical system from Agilent (Santa Clara, CA, USA). The chlorhexidine was analytically separated using a Zorbax Eclipse Plus C18 column (3.0 mm × 100 mm × 3.5 μm), and the column temperature was kept constant at 30 ± 0.5 °C. A mixture of 65% disodium hydrogen phosphate solution of 0.01 mol/L and 35% acetonitrile was used as the mobile phase. The UV detection was carried out at 254 nm, with an injection volume of 20 μL at a flow rate of 1.0 mL/min at 25 °C. The loading capacity (LC) (%) of chlorhexidine onto maghemite nanoparticles was calculated with the following formula [141]:

(2) $$LC\%=\frac{m_{CHX\text{-}NPS}}{m_{NPS}}\times 100\%$$

where $m_{CHX\text{-}NPS}$ is the weight of CHX within maghemite nanoparticles, and $m_{NPS}$ is the weight of the maghemite nanoparticles.

For investigating the release profile of CHX from maghemite NPSs’ surface, 50 mg of the CHX-NPS sample was mixed with 20 mL of deionized water from Merck Millipore (Milli-Q^®^ Integral Water Purification System, Darmstadt, Germany) at 37 °C for 4 h. The release profile of CHX was determined by UV Vis spectroscopy, at 254 nm, using a T70 UV/Vis spectrometer from PG Instruments (Leicestershire, UK).

### 4.3. In Vitro Toxicological Screening of the Maghemite NPSs, the Synthesized CHX-NPS Nanosystem, and the CHX Solution

#### 4.3.1. Reagents and Equipment

To perform the in vitro experiments, the following reagents were used: 2% chlorhexidine digluconate solution (Gluco Chex-In 2%) provided from CER- 83 KAMED (Stalowa Wola, Polonia); Eagle’s Minimum Essential Medium (EMEM—30-2003™); Dulbecco’s Modified Eagle’s Medium (DMEM—30-2002™); fetal bovine serum (FBS—30-2020™); penicillin/streptomycin antibiotic mixture; trypsin-EDTA solution purchased from the American Type Culture Collection (ATCC, Manassas, VA, USA); phosphate-buffered saline (PBS); the MTT (3-(4,5-dimethylthiazol2-yl)-2,5-diphenyltetrazolium bromide) kit, procured from Sigma-Aldrich (Merck KgaA, Darmstadt, Germany); and Hoechst 33342 dye from ThermoFisher Scientific (Waltham, MA, USA).

The devices used were Cytation 5 (plate reader) and Lionheart FX (automated microscope) provided by BioTek Instruments Inc. (Winooski, VT, USA) and the SteREO Discovery V8 stereomicroscope from ZEISS (Jena, Germany).

#### 4.3.2. Antimicrobial Activity

Through the broth dilution assay, the minimum inhibitory concentration (MIC) and minimum bactericidal concentration (MBC) were determined of the maghemite NPSs, the synthesized CHX-NPS nanosystem, and the CHX solution, according to the European Committee on Antimicrobial Susceptibility Testing (EUCAST) and the Clinical Laboratory and Standard Institute (CLSI). The protocol for broth dilution assay was described in detail in previous studies [142,143]. The microorganism strains used in the present study (*Staphylococcus aureus* (ATCC 25923), *Escherichia coli* (ATCC 25922), and *Pseudomonas aeruginosa* (ATCC 27853)), initially isolated on Columbia agar supplemented with 5% sheep blood (Thermo Scientific, Waltham, MA, USA), were purchased from the American Type Culture Collection (ATCC) (Manassas, VA, USA). For the dilution of the standardized bacterial inoculum of 0.5 McFarland until achieving approximately 5 × 10^5^ colony-forming units/mL (CFU), the NaCl 0.85% solution was used (bioMérieux, Marcy-l’Étoile, France). After that, the bacterial suspension and the test samples (maghemite NPSs, the CHX-NPS nanosystem, and CHX solution, at three different concentrations) were added to Mueller–Hinton broth (Thermo Scientific, Waltham, MA, USA). The MIC value, which represents the lowest concentration without visible growth, was interpreted after 24 h of incubation at 35 °C. Through the sub-cultivation of 1 μL suspension from the test tube, without visible growth, on Columbia agar with 5% sheep blood, the MBC value, which represents the lowest concentration suitable to kill 99.9% of the bacteria, was established. The determinations were performed in triplicate for each tested strain and each tested concentration of the test samples (maghemite NPSs, CHX-NPS nanosystem, and CHX solution).

#### 4.3.3. Cell Culture Conditions and Treatment Application

JB6 Cl 105 41-5a murine epidermal cells (CRL-2010™; ATCC, Manassas, VA, USA) and HaCaT human immortalized keratinocytes (300493; CLS, Eppelheim, Germany) were two healthy cell lines used for the in vitro evaluations. Each cell line was grown in a specific culture medium; thus, for the JB6 Cl 105 41-5a cell line, the specific culture medium used was EMEM supplemented with 5% FBS and 1% penicillin/streptomycin antibiotic mixture, while for the HaCaT cells, the specific culture medium was DMEM also supplemented with 10% FBS and 1% penicillin/streptomycin antibiotic mixture. Throughout the experiments, the cells were incubated under standard conditions (37 °C and 5% CO_2_).

After the cells’ preparation, they were exposed to three concentrations of interest samples: 10, 50, and 100 μg/mL maghemite NPSs, CHX-NPS nanosystem, and CHX solution, respectively. The cells were exposed to each test sample for 1 min and 2 min intervals, washed with PBS, and incubated for 24 hours (h) with the specific culture medium reported above. All the in vitro determinations were performed at a 24 h time point post-treatment.

#### 4.3.4. Cell Viability

The cell viability was assessed by the means of MTT (3-(4,5-dimethylthiazol-2-yl)-2,5-diphenyltetrazolium bromide) assay, at 24 h after exposure (1 and 2 min), to test samples (10, 50, 100 μg/mL maghemite NPSs, CHX-NPS nanosystem, and CHX solution). After 24 h, the old medium was replaced with 100 μL fresh medium and 10 μL of MTT reagent/well; then, the plates were incubated for a period of 3 h. After the incubation period, 100 μL/well of MTT solubilizing solution was added and the absorbance was read at 570 nm and 630 nm using Cytation 5.

#### 4.3.5. Nuclear Staining

Hoechst 33342 staining assay was applied to assess nuclear changes following the treatment with the highest and the lowest concentrations of test samples (10 and 100 μg/mL) at 24 h after exposure (1 and 2 min). The following protocol was conducted: (1) the Hoechst 33342 staining solution was diluted in PBS (1:2000); (2) the old culture medium was discarded; (3) 500 µL/well staining solution was added; and (4) the wells were incubated in a dark place for 5–10 min. After this time, the staining solution was removed and the cells were washed 3 times with PBS solution. Photographs of the cells were taken and analyzed using the Lionheart FX (automated microscope) device, Gen5™ Microplate Data Collection and Analysis Software (Version 3.14). The following formula was used to calculate the apoptotic index:

(3) $$\mathrm{AI}\;\left(\%\right)=\frac{Number\;of\;apoptotic\;cells}{Total\;number\;of\;cells}$$

### 4.4. In Ovo Toxicological Screening of the Maghemite NPSs, the Synthesized CHX-NPS Nanosystem, and the CHX Solution Through the HET-CAM (Hen’s Egg Test-Chorioallantoic Membrane) Assay

The irritant potential of maghemite NPSs, CHX-NPS nanosystem, and CHX solution was assessed using the HET-CAM assay on the 9th day of hen egg incubation. The highest concentration of each sample was chosen for this examination (100 μg/mL). The preparation of eggs was carried out according to the literature [144]. The embryonated eggs were kept in a special incubator at a temperature controlled daily (37 °C). For the HET-CAM test (day 9), test samples were prepared as follows: 100 μg/mL maghemite NPSs, 100 μg/mL CHX-NPS nanosystem, and 100 μg/mL CHX solution, together with 1% sodium dodecyl sulfate (SDS) as a positive control and distilled water (H_2_Od) as a negative control. A volume of 600 μL of each sample was used for the test and 5 min was monitored for potential changes (hemorrhage, lysis, and coagulation). Photographs of the membrane were taken both before the application of each sample (T0) and at the end of the 5 min (T5). The images were taken using the Discovery v.8 stereomicroscope and ZEN 174 core 3.8 software. The irritation score (IS) was calculated according to the following formula [145]:

(4) $$IS=5\times \frac{301-H}{300}+7\times \frac{301-L}{300}+9\times \frac{301-C}{300}$$

where IS is the index that indicates the irritant potential of test samples by measuring the time in which changes, such as hemorrhage—H, vascular lysis—L, or coagulation—C, occur on the chorioallantoic membrane in a 5 min time interval (300 s).

According to the results, the samples can be classified into one of three categories as follows: non-irritant if the IS is between 0 and 0.9; weak irritant if the IS is between 1 and 4.9; moderately irritant if the IS is between 5 and 8.9; and severely irritant if the IS is between 9 and 21 [146,147].

## 5. Conclusions

The current study undertook a safety profile investigation regarding the in vitro and in ovo screening of maghemite NPSs obtained through the combustion method, the chlorhexidine-carrier nanosystem based on iron oxide magnetic nanoparticles (CHX-NPS nanosystem), as well as CHX solution. Our findings strengthen the statement regarding the fact that the synthesized nano-carrier system composed of CHX solution incorporated onto iron oxide magnetic nanoparticles’ surface could be used in the dentistry domain, and the use of IOMNPs (maghemite) as drug carriers can be considered as an approach for oral pathogen therapy, due to their physicochemical features. As regards chlorhexidine, it remains a cornerstone in the prevention of infections in healthcare settings, particularly in the management of oral hygiene for critically ill patients, but ongoing research is necessary to optimize its use; address potential adverse effects, particularly in long-term applications; and ensure that healthcare providers are equipped with the knowledge to implement effective oral care protocols. Ongoing studies are focused on optimizing formulations that aim to enhance patient outcomes while minimizing the adverse effects of CHX, based on the synthesis and functionalization of IOMNPs to enhance their biocompatibility and reduce potential side effects associated with its use. The new formulation based on IOMNPs must provide effective antimicrobial action with reduced in vitro cytotoxicity on healthy cell lines. According to this statement, it was demonstrated in the present study that the absorption of chlorhexidine on the surface of magnetic nanoparticles is influenced by various factors, including nanoparticle size, surface chemistry, and the method of encapsulation. The integration of chlorhexidine with magnetic nanoparticles not only enhances its antimicrobial efficacy but also allows for controlled release, making it a promising approach for various medical applications. We demonstrate that the CHX-NPS nanosystem exhibited enhanced bacteriostatic and bactericidal properties against *E. coli*, *S. aureus*, and *P. aeruginosa*, as compared with those of the CHX solution, tested at the same concentrations as those of the synthesized nanosystem. In conclusion, the in vitro evaluation of chlorhexidine-carrier nanosystems utilizing iron oxide nanoparticles presents a promising strategy for enhancing the efficacy of this antimicrobial agent. The unique properties of IONPs, including their superparamagnetism and biocompatibility, make them ideal candidates for targeted drug delivery applications. Future research should focus on optimizing the formulation and delivery mechanisms of these nanosystems to maximize their therapeutic potential while ensuring safety and efficacy in clinical settings.

## Figures and Tables

**Table 1:** MIC and MBC of maghemite NPSs, CHX-NPS nanosystem, and CHX solution against *S. aureus*, *E. coli*, and *P. aeruginosa*.

Test Sample and Concentration	*S. aureus* (+) ATCC 25923		*E. coli* (−) ATCC 25922		*P. aeruginosa* (−) ATCC 27853	
	MIC (μg/mL) ± SD	MBC (μg/mL) ± SD	MIC (μg/mL) ± SD	MBC (μg/mL) ± SD	MIC (μg/mL) ± SD	MBC (μg/mL) ± SD
**Maghemite NPSs**						
10 μg/mL	>800 ± 10.422	>800 ± 10.422	>800 ± 10.422	>800 ± 10.422	>800 ± 10.422	>800 ± 10.422
50 μg/mL	349 ± 7.071	433 ± 7.071	288 ± 6.248	292 ± 6.248	304 ± 7.302	373 ± 7.302
100 μg/mL	110 ± 2.312	130 ± 2.312	80 ± 2.097	100 ± 2.097	90 ± 2.098	110 ± 2.098
**CHX-NPS nanosystem**						
10 μg/mL	19 ± 0.050	20 ± 0.050	12 ± 0.050	14 ± 0.050	24 ± 0.050	24 ± 0.050
50 μg/mL	8 ± 0.013	9 ± 0.013	6 ± 0.012	7 ± 0.012	10 ± 0.014	15 ± 0.014
100 μg/mL	3 ± 0.012	4 ± 0.012	3 ± 0.012	3 ± 0.012	5 ± 0.013	7 ± 0.013
**CHX solution**						
10 μg/mL	43 ± 0.056	45 ± 0.056	30 ± 0.051	31 ± 0.051	81 ± 0.067	86 ± 0.067
50 μg/mL	21 ± 0.041	23 ± 0.041	16 ± 0.041	18 ± 0.041	48 ± 0.045	48 ± 0.045
100 μg/mL	6 ± 0.013	7 ± 0.013	3 ± 0.012	4 ± 0.012	10 ± 0.020	11 ± 0.020

**Table 2:** The statistical significance (*p*-values) of the cell viability results were obtained 24 h after exposure of JB6 Cl 41-5a cells to 1 and 2 min of NPSs, CHX-NPS, and CHX (10, 50, 100 μg/mL).

Sample	Period of Exposure	*p*-Values
Control	1 min	-
NPS 10 μg/mL		<0.05 *
NPS 50 μg/mL		NS
NPS 100 μg/mL		NS
CHX-NPS 10 μg/mL		NS
CHX-NPS 50 μg/mL		NS
CHX-NPS 100 μg/mL		≤0.0001 ****
CHX 10 μg/mL		NS
CHX 50 μg/mL		≤0.0001 ****
CHX 100 μg/mL		≤0.0001 ****
Control	2 min	-
NPS 10 μg/mL		NS
NPS 50 μg/mL		NS
NPS 100 μg/mL		NS
CHX-NPS 10 μg/mL		<0.05 *
CHX-NPS 50 μg/mL		NS
CHX-NPS 100 μg/mL		≤0.0001 ****
CHX 10 μg/mL		NS
CHX 50 μg/mL		≤0.0001 ****
CHX 100 μg/mL		≤0.0001 ****

NS—not significant. ‘*’ indicates statistical significance (* *p* < 0.05 and **** *p* ≤ 0.0001).

**Table 3:** The statistical significance (*p*-values) of the cell viability results were obtained 24 h after exposure of HaCaT cells to 1 and 2 min of NPSs, CHX-NPS, and CHX (10, 50, 100 μg/mL).

Sample	Period of Exposure	*p*-Values
Control	1 min	-
NPS 10 μg/mL		NS
NPS 50 μg/mL		NS
NPS 100 μg/mL		NS
CHX-NPS 10 μg/mL		<0.001 ***
CHX-NPS 50 μg/mL		≤0.0001 ****
CHX-NPS 100 μg/mL		≤0.0001 ****
CHX 10 μg/mL		<0.01 **
CHX 50 μg/mL		≤0.0001 ****
CHX 100 μg/mL		≤0.0001 ****
Control	2 min	-
NPS 10 μg/mL		NS
NPS 50 μg/mL		NS
NPS 100 μg/mL		NS
CHX-NPS 10 μg/mL		NS
CHX-NPS 50 μg/mL		NS
CHX-NPS 100 μg/mL		≤0.0001 ****
CHX 10 μg/mL		≤0.0001 ****
CHX 50 μg/mL		≤0.0001 ****
CHX 100 μg/mL		≤0.0001 ****

NS—not significant. ‘*’ indicates statistical significance (** *p* < 0.01; *** *p* < 0.001; and **** *p* ≤ 0.0001).

**Table 4:** The statistical significance (*p*-values) of apoptotic index results obtained 24 h after JB6 Cl 41-5a cells were exposed to 1 and 2 min of NPSs, CHX-NPS, and CHX (10 and 100 μg/mL).

Sample	Period of Exposure	*p*-Values
Control	1 min	-
NPS 10 μg/mL		NS
NPS 100 μg/mL		<0.05 *
CHX-NPS 10 μg/mL		NS
CHX-NPS 100 μg/mL		≤0.0001 ****
CHX 10 μg/mL		<0.05 *
CHX 100 μg/mL		≤0.0001 ****
Control	2 min	-
NPS 10 μg/mL		NS
NPS 100 μg/mL		<0.01 **
CHX-NPS 10 μg/mL		<0.001 ***
CHX-NPS 100 μg/mL		≤0.0001 ****
CHX 10 μg/mL		≤0.0001 ****
CHX 100 μg/mL		≤0.0001 ****

NS—not significant. ‘*’ indicates statistical significance (* *p* < 0.05; ** *p* < 0.01; *** *p* < 0.001; and **** *p* ≤ 0.0001).

**Table 5:** The statistical significance (*p*-values) of apoptotic index results obtained 24 h after exposure of HaCaT cells to 1 and 2 min of NPSs, CHX-NPS, and CHX (10 and 100 μg/mL).

Sample	Period of Exposure	*p*-Values
Control	1 min	-
NPS 10 μg/mL		NS
NPS 100 μg/mL		NS
CHX-NPS 10 μg/mL		NS
CHX-NPS 100 μg/mL		≤0.0001 ****
CHX 10 μg/mL		≤0.0001 ****
CHX 100 μg/mL		≤0.0001 ****
Control	2 min	-
NPS 10 μg/mL		NS
NPS 100 μg/mL		NS
CHX-NPS 10 μg/mL		≤0.0001 ****
CHX-NPS 100 μg/mL		≤0.0001 ****
CHX 10 μg/mL		≤0.0001 ****
CHX 100 μg/mL		≤0.0001 ****

NS—not significant. ‘*’ indicates statistical significance (**** *p* ≤ 0.0001).

**Table 6:** Irritant score (IS) for maghemite NPS 100 μg/mL, CHX-NPS nanosystem 100 μg/mL, and CHX solution 100 μg/mL using the HET-CAM assay. H_2_Od-negative control and SDS 1%-positive control.

Test Sample	IS	Irritation Category
H_2_Od	0.069	Non-irritant
SDS 1%	20.129	Severely irritant
NPS 100 μg/mL	0.069	Non-irritant
CHX-NPS 100 μg/mL	0.069	Non-irritant
CHX 100 μg/mL	7.826	Irritant

**Table 7:** Characteristics of γ-Fe_2_O_3_ NPSs before (A) and after (B) the treatment with H_2_O_2._

Treatment	XRD	D_XRD_ [nm]	S_BET_ [m^2^/g]	D_BET_ [nm]	M_s_ [emu/g]
A—before H_2_O_2_ treatment	γ-Fe_2_O_3_	5	3.1	372	6.5
B—after H_2_O_2_ treatment	γ-Fe_2_O_3_	5	192	6	23

## Data Availability

All the data obtained are included in the present article. Further inquiries can be directed to the corresponding author.
